# SAP30 deacetylates the Tas protein to inhibit PFV replication

**DOI:** 10.1186/s13578-025-01400-2

**Published:** 2025-04-24

**Authors:** Chenchen Wang, Junshi Zhang, Yali Xu, Jiawei Zhao, Manman Qiu, Xingli Zhao, Guoqiang Li, Wentao Qiao, Juan Tan

**Affiliations:** 1 Key Laboratory of Molecular Microbiology and Technology, College of Life Sciences, Ministry of Education, Nankai University, Tianjin, 300071 China; 2 Department of Hematology, Oncology Center, Tianjin Union Medical Center, The First Affiliated Hospital of Nankai University, Tianjin, 300121 China; 3 Tianjin Cancer Institute of Integrative Traditional Chinese and Western Medicine, Tianjin Union Medical Center, The First Affiliated Hospital of Nankai University, Tianjin, 300121 China

**Keywords:** Prototype foamy virus, SAP30, Tas, Transcription, Deacetylation

## Abstract

**Background:**

Foamy viruses (FVs), a unique class of retroviruses, establish lifelong latent infections in the host without causing symptoms, contributing to the relatively slow progress in FV research. However, key mutations in FVs can result in severe consequences due to their broad cellular tropism, underscoring the importance of studying latent FV infections.

**Results:**

To identify new host proteins involved in the replication of prototype foamy virus (PFV), we previously infected the human fibrosarcoma cell line HT1080 with PFV and performed transcriptomic sequencing. The analysis revealed a significant upregulation of *SAP30* mRNA levels following PFV infection. Further experiments demonstrated that PFV infection enhances *SAP30* promoter activity via the Tas protein, leading to increased *SAP30* mRNA and protein expression. Overexpression of SAP30 inhibited PFV replication, whereas knockdown of endogenous SAP30 enhanced PFV replication. Furthermore, SAP30 interacted with the Tas protein to induce its deacetylation, thereby suppressing Tas-mediated transactivation of the PFV LTR and IP promoters. The Sin3 interaction domain at the C-terminus of SAP30 was identified as the critical domain for inhibiting PFV transcription.

**Conclusions:**

Our findings suggest that SAP30 inhibits PFV replication by deacetylating the Tas protein, thereby disrupting its transcriptional activation function. **Key words**: prototype foamy virus; SAP30; Tas; transcription; deacetylation.

## Introduction

Foamy viruses (FVs) are complex and unique retroviruses that induce severe cytopathic effects in vitro but establish long-term latent infections within hosts [1, 2]. The prototype foamy virus (PFV) is the only known FV capable of interspecies transmission to humans, although it remains nonpathogenic in humans [3, 4]. In addition to encoding the structural proteins Gag, Pol and Env, PFV also encodes two non-structural proteins, Tas and Bet [5–8]. The PFV Tas protein, a transcriptional regulator with a molecular weight of approximately 36 kDa, contains a DNA-binding domain (DNA-BD) and a C-terminal transcription activation domain (AD) [9].

As a transcriptional regulator, Tas directly interacts with the PFV long terminal repeat (LTR) and internal promoter (IP) to regulate gene expression [10, 11]. Host factors also interact with Tas to modulate this transcriptional process, with acetylation shown to enhance its transcriptional activation function. Studies have demonstrated that PFV Tas associates with the histone acetyltransferases P300 and PCAF, whose overexpression enhances Tas-mediated transcriptional activation [12, 13]. Our laboratory’s research similarly revealed that P300 induces the acetylation of the bovine foamy virus (BFV) BTas protein [14]. Additionally, the Tas protein of feline foamy virus (FFV) is also acetylated by PCAF [13]. These findings suggest that the acetylation of foamy virus Tas proteins is essential for their transcriptional activation function.

SAP30 is a core component of the human histone deacetylase (HDAC) complex, playing a crucial role in stabilizing the Sin3/HDAC complex to regulate the transcription of cellular genes [15]. The SAP30 protein is located in the cell nucleus and consists of a low-conserved N-terminal region, a highly conserved central region and a C-terminal Sin3 interaction domain [16, 17]. The amino acid residues 129–220 at the C-terminus are critical and sufficient for SAP30 binding to the Sin3/HDAC complex [17]. SAP30 exerts antiviral effects through interactions with transcriptional repressors. For example, the papillomavirus binding factor (PBF) acts as a repressor of human papillomavirus (HPV) transcription. Although PBF binds to GC-rich sequence elements in the HPV upstream promoter, its repressive effect relies on interaction with SAP30, which recruits the Sin3/HDAC complex to inhibit viral gene transcription via HDAC activity [18]. Additionally, following herpes simplex virus type 1 (HSV-1) infection in human fibroblasts, the expression of human transcription regulator protein (HTRP) is upregulated. This upregulated HTRP interacts with SAP30 to enhance HDAC activity, thereby synergistically suppressing HSV-1 gene transcription and viral replication [19].

In this study, we found that PFV infection enhances SAP30 promoter activity via the Tas protein, leading to elevated SAP30 mRNA and protein levels. SAP30 interacts with the Tas protein, deacetylates it and attenuates its ability to activate PFV LTR and IP promoters, thereby inhibiting PFV replication. The C-terminal 129–220 aa of SAP30 is the key domain for its inhibitory function.

## Materials and methods

### RNA-seq and data analysis

HT1080 cells were infected with PFV for 6 h, 12 h, and 24 h, with uninfected cells serving as controls. Total RNA was extracted using the TRIzol reagent, and RNA concentration and purity were assessed using a NanoDrop UV spectrophotometer. Following quality confirmation, the RNA samples were sent to the Beijing Genomics Institute for high-throughput sequencing on the Illumina HiSeq platform. Raw image data were converted into nucleotide sequences through base calling, generating raw reads in FASTQ format. FastQC was used to assess data quality, and low-quality reads were filtered, the resulting high-quality clean reads were subjected to downstream analysis. Sequence alignment and quantification were performed, and gene and transcript expression levels were normalized using the FPKM method. Differentially expressed genes were identified using the PoissonDis algorithm, with thresholds set at a false discovery rate (FDR) ≤ 0.001 and a fold change (FC) ≥ 1, allowing for the comparison of gene expression between infected and uninfected groups.

### Plasmid constructs

Human SAP30 cDNA was cloned into the pCMV-3HA vector (Clontech, Mountain View, CA, USA). SAP30 truncations were constructed based on pCMV-3HA-SAP30. LTR-luc [20], IP-luc [20] and PFV full-length infectious clone (pcPFV) [21] were generously provided by Maxine L. Linial (Division of Basic Sciences, Fred Hutchinson Cancer Research Center, Seattle, WA, USA). 3.1-Tas [22] and Flag-Tas [22] were constructed as described previously. Myc-Tas was constructed by inserting the cDNA of Tas into pCMV-Tag-3B. HA-P300 was purchased from Upstate Biotechnology (Lake Placid, NY, USA). pGL-3162 was constructed by inserting the *SAP30* promoter into pGL3-basic vector. pCMV-β-Gal was purchased from Invitrogen (Carlsbad, CA, USA). Cells were transfected using PEI transfection reagent (Polysciences, Warrington, PA, USA) according to the manufacturer’s instructions [20].

### Antibodies

The following antibodies were used in this study: polyclonal rabbit anti-SAP30 and anti-acetyl-lysine antibodies (Abcam, Cambridge, MA, USA); monoclonal mouse anti-HA, rabbit anti-HA, monoclonal mouse anti-Flag, rabbit anti-Flag, monoclonal mouse anti-Myc, and monoclonal mouse anti-Tubulin (Sigma-Aldrich, St. Louis, MO, USA); mouse anti-goat IgG-horseradish peroxidase (HRP) (Santa Cruz Biotechnology, CA, USA); rabbit anti-goat IgG-HRP (Cell Signaling Technology, Danvers, MA, USA); fluorescein isothiocyanate (FITC)- and rhodamine (TRITC)-conjugated secondary antibodies (Jackson ImmunoResearch Laboratories); and polyclonal mouse anti-PFV Gag, which was prepared in our laboratory [22].

### Cell culture and virus infection

HEK293T, HeLa and HT1080 cells were cultured in a cell culture incubator at 37℃ with 5% CO_2_. The culture medium contained 10% (v/v) fetal bovine serum and was supplemented with penicillin and streptomycin. Cells were passaged every 2 to 3 days based on their growth status. HEK293T cells were transfected with the PFV infectious clone pcPFV. After 48 h, the supernatant was collected and centrifuged at 1,000 g for 3 min to prepare the virus stock. HT1080 cells were infected with the PFV stock, after 48 h, the supernatant and 1/10 of the cells were collected to infect the PFV indicator cell line PFVL. Luciferase activity was measured to indicate the virus titer.

### Generation of knockdown cell lines

To knock down endogenous SAP30 using shRNA, the following target sequences were used: shSAP30#1, 5′-GATCCGCCAGAGGTTGATTTATACCAATTCAAGAGATT GGTATAAATCAACCTCTGGTTTTTTACGCGTG-3′ (forward) and 5′-AATTCACG CGTAAAAAACCAGAGGTTGATTTATACCAATCTCTTGAATTGGTATAAATCAACCTCTGGCG-3′ (reverse); shSAP30#2, 5′-GATCCGCACAACTTGTTGAGATAG TTTTCAAGAGAAACTATCTCAACAAGTTGTGCTTTTTTACGCGTG-3′ (forward) and 5′-AATTCACGCGTAAAAAAGCACAACTTGTTGAGATAGTTTCTCT TGAAAACTATCTCAACAAGTTGTGCG-3′ (reverse); shSAP30#3, 5′-GATCCGG ATAAGAGCGCAAGGCATCTTTCAAGAGAAGATGCCTTGCGCTCTTATCCTTTTTTACGCGTG-3′ (forward) and 5′-AATTCACGCGTAAAAAAGGATAAGAG CGCAAGGCATCTTCTCTTGAAAGATGCCTTGCGCTCTTATCCG-3′ (reverse). HEK293T cells were transfected with shRNA, MLV Gag-pol and VSV-G. After 48 h, the culture medium was collected and centrifuged at 3,000 rpm for 3 min. The supernatant was then used to infect HT1080 cells. After another 48 h, puromycin (2 µg/mL) was added to select cells with stable SAP30 knockdown.

### Real-time PCR

Cells were lysed using TRIzol reagent (Invitrogen, Carlsbad, CA, USA) and RNA was extracted according to the manufacturer’s protocol. The RNA was then reverse transcribed into cDNA, which was used as a template for real-time PCR. The following primers were used: SAP30, 5′-GAGCTGGATAAGAGCGCAAGG-3′ (forward) and 5′-AGACCTAAAGTGGCAACCAAC-3′ (reverse); GAPDH, 5′-AACAGCGACAC CCACTCCTC-3′ (forward) and 5′-CATACCAGGAAATGAGCTTGACAA-3′ (reverse). The 2^−ΔΔCt^ method was used for data analysis.

### Western blotting

Cells were collected and lysed with lysis buffer on ice for 30 min and then centrifuged at 12,000 rpm for 10 min at 4℃. The supernatant was mixed with loading buffer and boiled at 100℃ for 15 min. Samples were separated by SDS-PAGE and transferred to polyvinylidene difluoride (PVDF) membranes (GE Healthcare, Chicago, IL, USA). The membranes were blocked with 5% nonfat milk for 45 min and then incubated with primary antibodies for 1.5 h and secondary antibodies for 45 min. Protein signals were detected by chemiluminescence (Merck Millipore, Darmstadt, Germany).

### Luciferase reporter assay

Cells were harvested and lysed with 1×luciferase lysis buffer for 15 min at room temperature. The lysates were then centrifuged at 12,000 rpm for 3 min, and the supernatants were collected for the assay. Luciferase activity was measured using a luciferase reporter assay system kit (Promega, Madison, WI, USA) according to the manufacturer’s instructions.

### Alu polymerase chain reaction

HT1080 cells were transfected with SAP30 or its empty vector and infected with PFV stock 24 h later. As positive controls, the reverse transcriptase inhibitor AZT (10 µM) [23] and the integrase inhibitor Raltegravir (10 µM) [24] were added 2 h before infection. After 48 h, cells were harvested and total DNA was extracted. The integrated PFV provirus genome was detected by semi-quantitative PCR using the Alu-PCR method, as previously described [25].

### Immunofluorescence assay

HeLa cells were plated on coverslips and fixed with 4% formaldehyde for 10 min at room temperature. Following fixation, the cells were washed with PBS and permeabilized with 0.1% Triton X-100 for 10 min, then washed four times with PBS. After blocking with 5% nonfat milk for 45 min, cells were incubated with primary antibodies for 2 h and fluorescein isothiocyanate (FITC)- or tetramethylrhodamine isothiocyanate (TRITC)-conjugated secondary antibodies for 40 min. The samples were then stained with 4’,6-diamidino-2-phenylindole (DAPI, 1:2,000) and imaged using an Olympus IX71 fluorescence microscope (Olympus, Tokyo, Japan).

### Coimmunoprecipitation

After 48 h of plasmid transfection, cells were harvested and centrifuged at 1,800 rpm for 3 min at room temperature. The supernatant was removed and cells were resuspended in PBS and then centrifuged at 2,800 rpm for 3 min at 4℃. Cells were lysed using IP lysis Buffer (50 mM pH 8.0 Tris HCL, 150 mM NaCl, 1% NP40, 50×cocktail) and centrifuged at 12,000 rpm for 10 min at 4℃. Part of the supernatant was added to loading buffer, and the rest was incubated with antibodies at 4 °C for 2.5 h. The mixture was then combined with Protein A beads and incubated for 3 h at 4℃. After mixing, the samples were washed six times with IP lysis buffer and boiled at 100 °C for 20 min. The samples were then stored at -20 °C or used for Western blotting to detect target protein interaction.

## Results

### **PFV infection upregulates***SAP30*

**Fig. 1:**
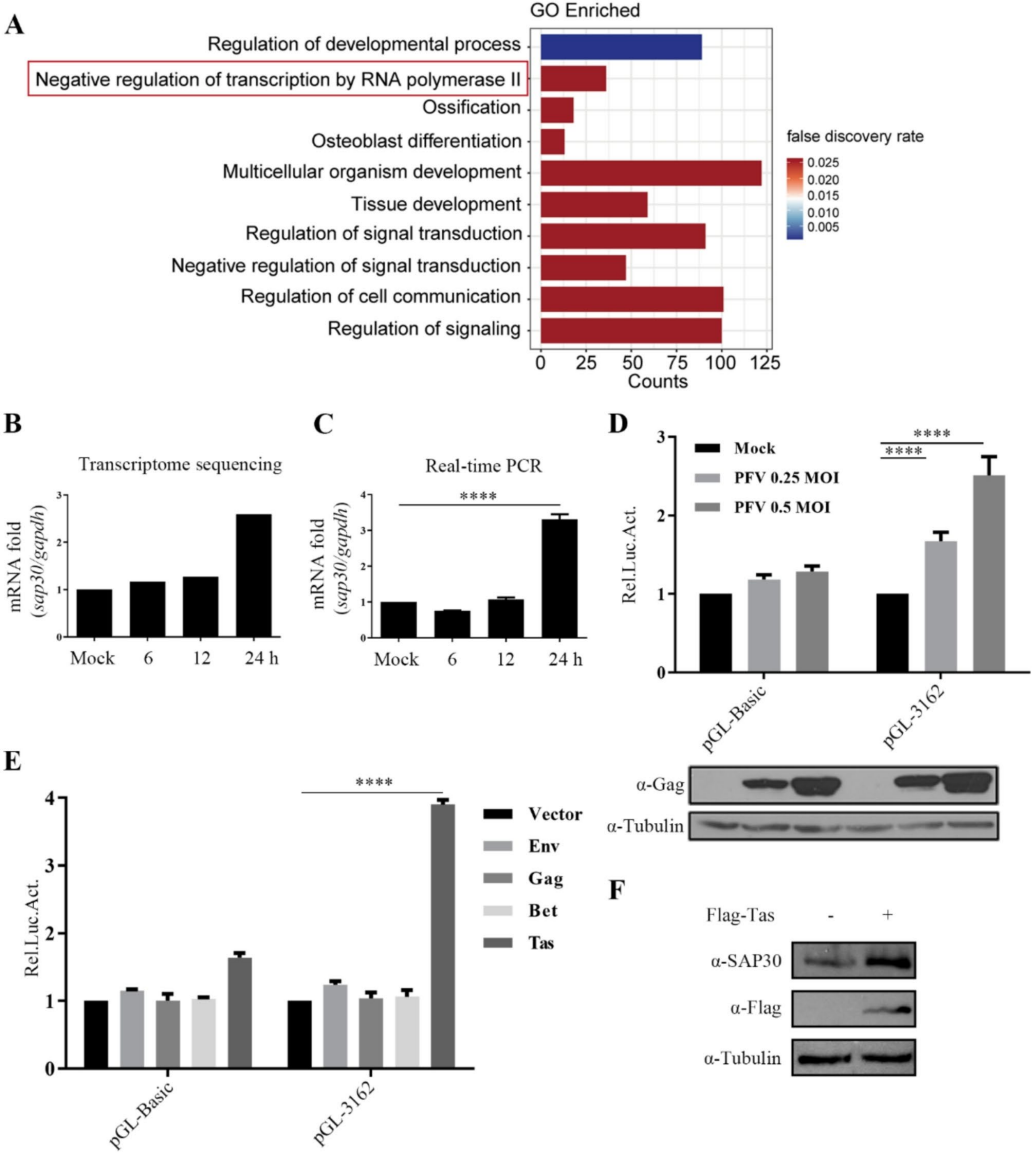
PFV infection upregulates *SAP30*. (**A**) HT1080 cells were infected with PFV for 6 h, 12 h, and 24 h. Total RNA was extracted for transcriptomics sequencing. GO enrichment analysis from the PPI network of differentially expressed genes generated via the STRING database. The top 10 enriched GO biological processes are shown, with the second-ranked term, “negative regulation of RNA polymerase II-mediated transcription,” highlighted in red. (**B**) The result of transcriptomic sequencing. (**C**) HT1080 cells were infected with PFV for 6 h, 12 h, and 24 h. Total RNA was extracted and reverse transcribed into cDNA. Real-time PCR was performed with gene-specific primers to detect the mRNA level of *SAP30*. (**D**) HT1080 cells were transfected with pGL-3162 (a recombinant plasmid containing the SAP30 promoter) or pGL3-basic (a luciferase reporter vector lacking promoter sequences, function as a negative control). Twenty-four hours post-transfection, cells were infected with PFV (MOI = 0.25 or 0.5). Luciferase activities were measured and normalized to β-gal catalytic activities. (**E**) HEK293T cells were transfected with pGL-3162 or empty vector, along with Flag-Env, Flag-Gag, Myc-Bet, 3.1-Tas and pCMV-β-gal. Forty-eight hours post-transfection, luciferase activities were measured and normalized to β-gal catalytic activities. (**F**) HEK293T cells were transfected with Flag-Tas or empty vector, and SAP30 expression was detected by Western blotting 48 h post-transfection

To further investigate the cellular factors involved in PFV replication, HT1080 cells were infected with PFV stock for 6, 12, and 24 h, followed by RNA extraction for transcriptomic sequencing (GSE200199). Analysis of the sequencing data revealed that after 24 h of PFV infection, the mRNA levels of 294 cellular factors were significantly upregulated, while 100 cellular factors were significantly downregulated [26]. To identify key transcriptional regulators, 394 differentially expressed genes (DEGs) were analyzed using the STRING database (v11.5, https://string-db.org/) to construct a protein-protein interaction (PPI) network. Functional enrichment analysis of the network identified the top 10 significantly enriched Gene Ontology (GO) biological processes (false discovery rate < 0.05). Among these, the category “negative regulation of RNA polymerase II-mediated transcription” ranked second, containing 36 genes, including known antiviral regulators such as ATF3 and HDAC4 (Fig. 1A).

SAP30, a core component of the Sin3/HDAC complex, mediates transcriptional repression via histone deacetylation. Although SAP30 has been reported to regulate the replication of HPV and HSV-1, its interaction with retroviruses remains unclear. Therefore, SAP30 was selected as the focus of this study. The transcriptomic sequencing results (Fig. 1B) were validated by real-time PCR, confirming a significant increase in *SAP30* mRNA levels after 24 h of PFV infection (Fig. 1C). Since promoters regulate gene transcription, we examined the effect of PFV infection on *SAP30* promoter activity and observed a dose-dependent upregulation of its basal transcriptional activity (Fig. 1D). Further experiments demonstrated that the PFV Tas protein enhanced *SAP30* promoter activity and increased SAP30 protein expression (Fig. 1E and F). These results suggest that PFV enhances *SAP30* promoter activity through the Tas protein, leading to increased *SAP30* mRNA and protein levels.

### SAP30 inhibits PFV replication

**Fig. 2:**
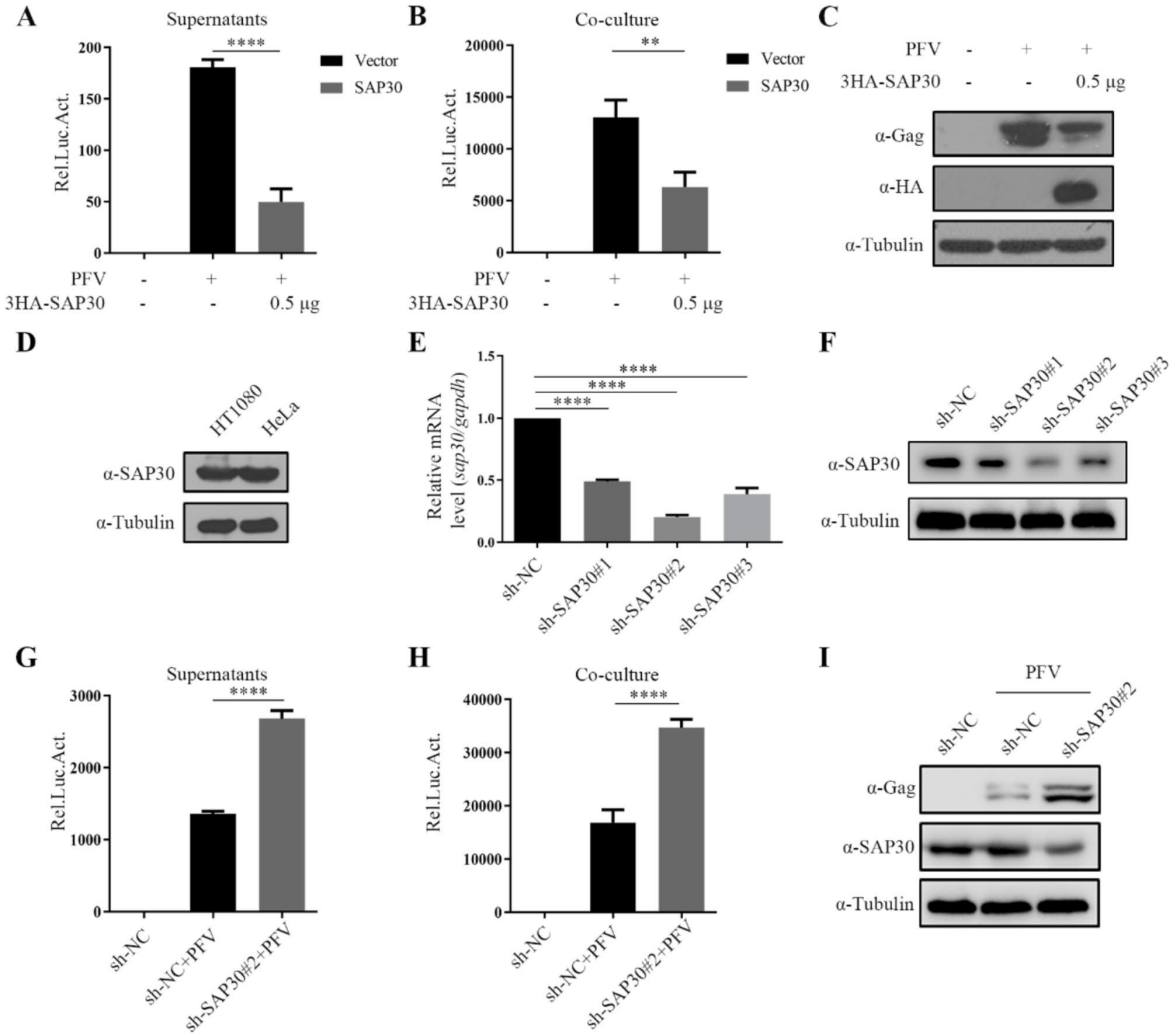
SAP30 inhibits PFV replication. (**A**–**C**) HT1080 cells were transfected with SAP30 or empty vector. Twenty-four hours post-transfection, cells were infected with PFV (MOI = 0.5). After 48 h, 600 µl of supernatants (**A**) or 1/10 infected HT1080 cells (**B**) were co-cultured with PFVL cells. The luciferase activity was measured 48 h later, and the remaining HT1080 cells were lysed for Western blotting (**C**). (**D**) Equal amounts of HT1080 and HeLa cells were lysed for Western blotting. (**E** and **F**) sh-Control and sh-SAP30 cells were collected, real-time PCR (**E**) and Western blotting (**F**) were performed to detect SAP30 expression levels. (**G–I**) Control and sh-SAP30#2 cell lines were infected with 0.5 MOI PFV. Forty-eight hours post-infection, 600 µl of supernatants (**G**) or 1/10 infected HT1080 cells (**H**) were co-cultured with PFVL cells. The luciferase activity was measured 48 h later, and the remaining HT1080 cells were lysed for Western blotting (**I**)

Next, we investigated the impact of SAP30 on PFV replication. To assess the effect of SAP30 overexpression, HT1080 cells were transfected with SAP30 and subsequently infected with PFV stock. After 48 h, the culture supernatant and cells were co-cultured with the PFV indicator cell line PFVL. Viral titers and protein expression were evaluated using luciferase activity and Western blotting, respectively. As shown in Fig. 2A–C, SAP30 overexpression significantly reduced PFV replication compared to the control, indicating that SAP30 inhibits PFV replication. We then examined the effect of endogenous SAP30 on PFV replication. Figure 2D shows that endogenous SAP30 expression levels are similar in HT1080 and HeLa cells. Since PFV exhibits stronger replication ability in HT1080 cells, we chose HT1080 to screen cell lines that knock down SAP30. The results indicated that knockdown cell line #2 was the most effective (Fig. 2E and F). Using this knockdown line, we observed a significant increase in PFV replication upon SAP30 knockdown (Fig. 2G–I), further confirming that SAP30 inhibits PFV replication.

### SAP30 inhibits PFV transcription

**Fig. 3:**
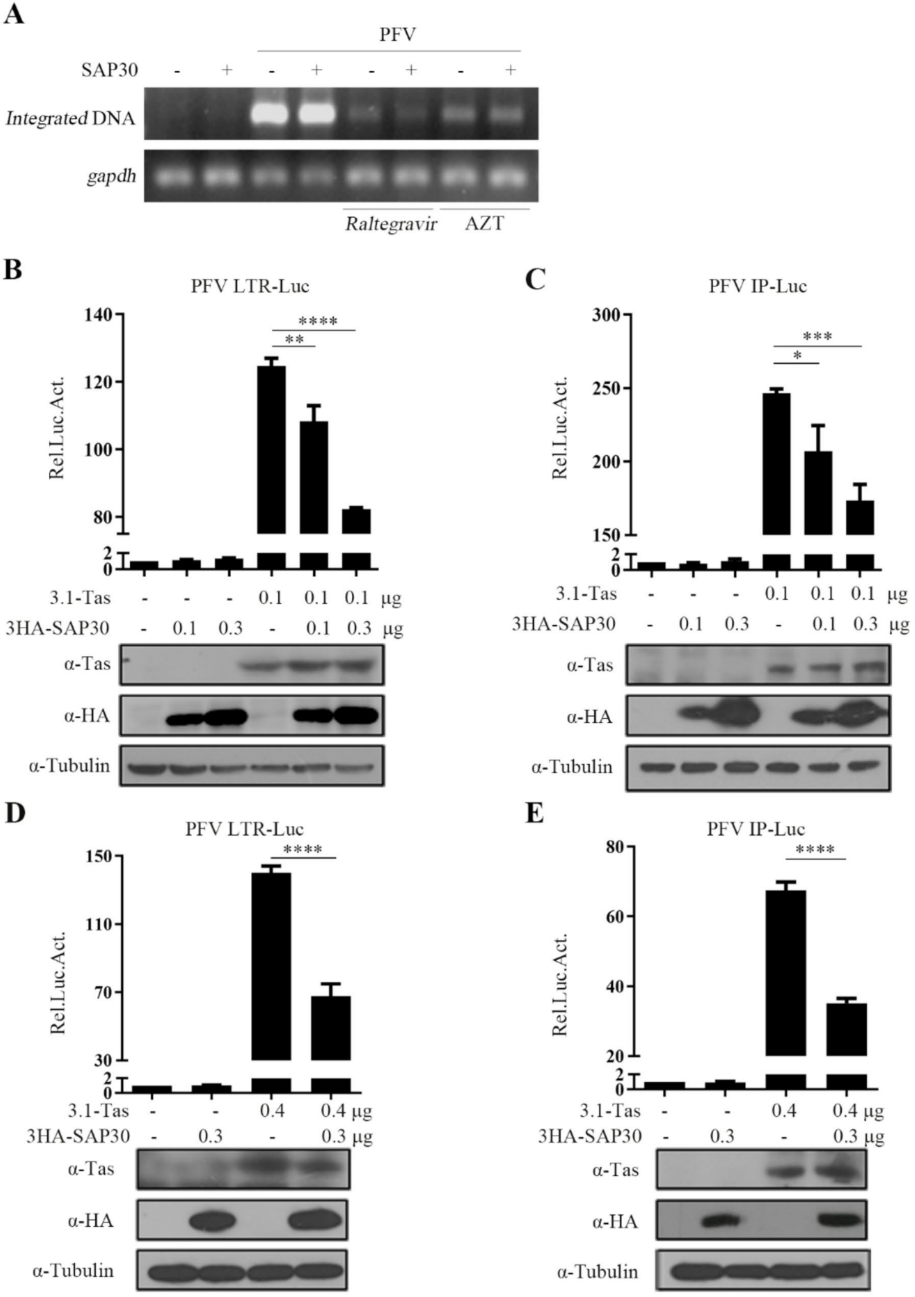
SAP30 inhibits PFV transcription. (**A**) The level of proviral DNA integration was detected by Alu-PCR, as described in the Materials and Methods. (**B** and **C**) Co-transfection of LTR-Luc or IP-Luc with 3.1-Tas and different amounts of 3HA-SAP30 in HEK293T cells. pCMV-β-gal was also transfected to normalize transfection efficiency. Forty-eight hours post-transfection, luciferase activities were measured and corrected by β-gal catalytic activities. Remaining cell lysate was used for Western blotting. (**D** and **E**) The same experiment was repeated in HT1080 cells

After confirming the inhibitory effect of SAP30 on PFV replication, we next investigated which stage of the PFV life cycle is affected by SAP30. The PFV life cycle is divided into early and late replication stages with integration as the dividing line [3, 27]. To examine whether SAP30 influences PFV integration, we employed Alu-PCR [28] to detect the integration of PFV genome. As shown in Fig. 3A, both the integrase inhibitor Raltegravir and the reverse transcriptase inhibitor AZT significantly inhibit PFV integration, whereas SAP30 overexpression had no effect. These results indicate that SAP30 does not influence the early replication stage of PFV. We then examined whether SAP30 affects the late replication stage. The Tas protein activates transcription by binding to the PFV LTR and IP promoters. To investigate the effect of SAP30 on PFV transcription, we performed experiments using LTR-Luc and IP-Luc reporter plasmids. As shown in Fig. 3B and C, SAP30 did not significantly alter the basal transcriptional activity of the PFV LTR and IP promoters. However, it inhibited Tas-mediated transactivation of these promoters in a dose-dependent manner. Further experiments in HT1080 cells confirmed that SAP30 suppresses the transactivation function of the PFV Tas protein (Fig. 3D and E). Collectively, these results suggest that SAP30 inhibits PFV transcription.

### SAP30 interacts with the PFV Tas protein

**Fig. 4:**
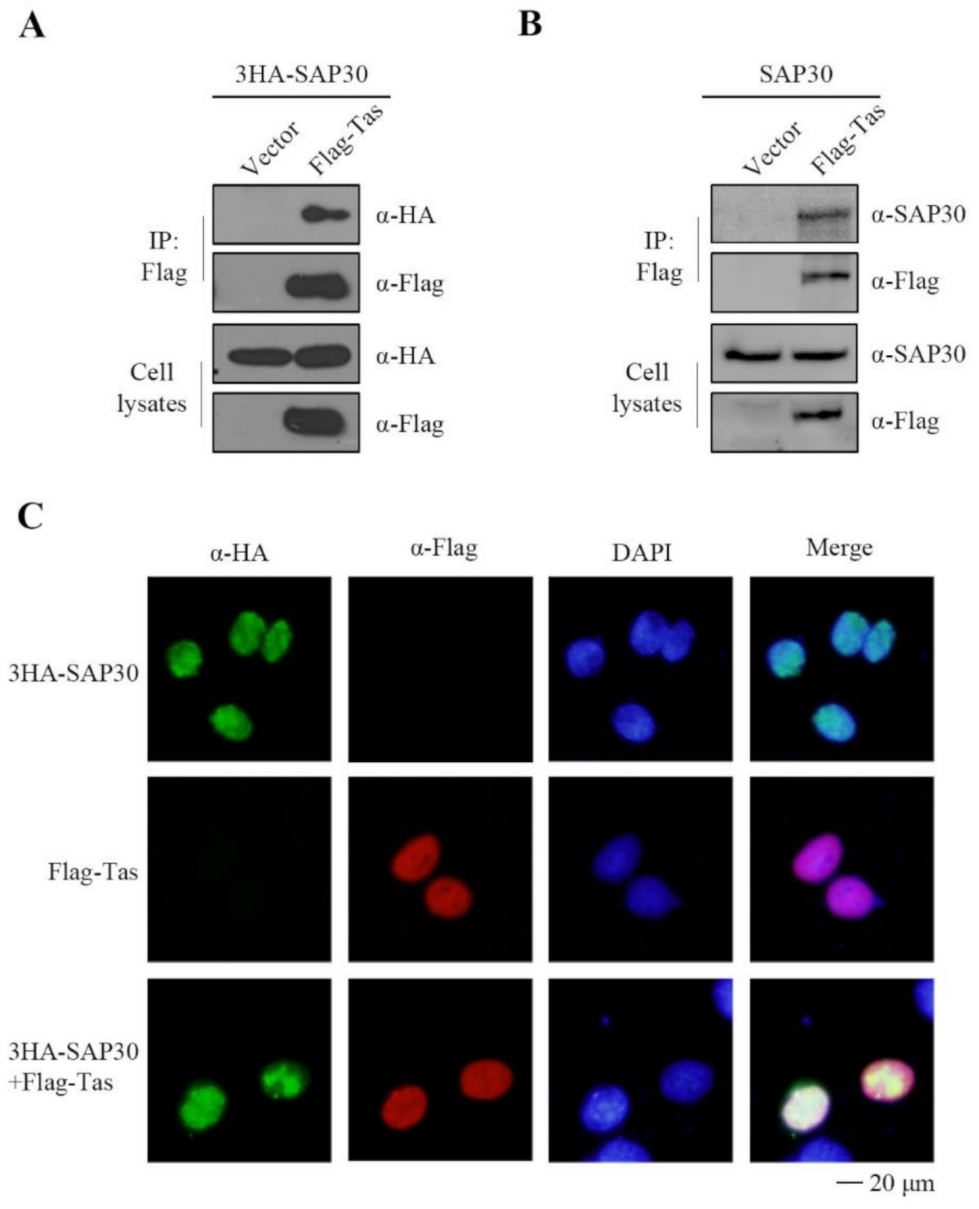
SAP30 interacts with the PFV Tas protein. (**A**) HEK293T cells were transfected with 3HA-SAP30 and empty vector or Flag-Tas. Forty-eight hours post-transfection, co-immunoprecipitation was performed with Flag antibodies. Western blot analysis of samples from cell lysates and immunoprecipitates using HA and Flag antibodies. (**B**) HEK293T cells were transfected with Flag-Tas or empty vector. Forty-eight hours post-transfection, co-immunoprecipitation was performed with Flag antibodies. Western blot analysis of samples from cell lysates and immunoprecipitates using SAP30 and Flag antibodies. (**C**) HeLa cells were transfected with 3HA-SAP30 or Flag-Tas or both. Forty-eight hours post-transfection, indirect immunofluorescence assay (IFA) was used to localize SAP30 (with HA antibodies and tetramethyl rhodamine isocyanate [FITC]-conjugated secondary antibodies) and Tas (with Flag antibodies and fluorescein isothiocyanate [TRITC]-conjugated secondary antibodies). Nuclei were visualized using DAPI staining

Since SAP30 inhibits PFV replication by targeting the Tas protein, we performed co-immunoprecipitation assays to examine their interaction. As shown in Fig. 4A and B, both overexpressed and endogenous SAP30 interacted with the Tas protein. Immunofluorescence analysis of SAP30 and Tas subcellular localization revealed that both proteins were predominantly localized in the nucleus (Fig. 4C). These findings indicate that SAP30 co-localizes and interacts with Tas in the nucleus.

### SAP30 Sin3 interaction domain is essential for PFV transcriptional Inhibition

**Fig. 5:**
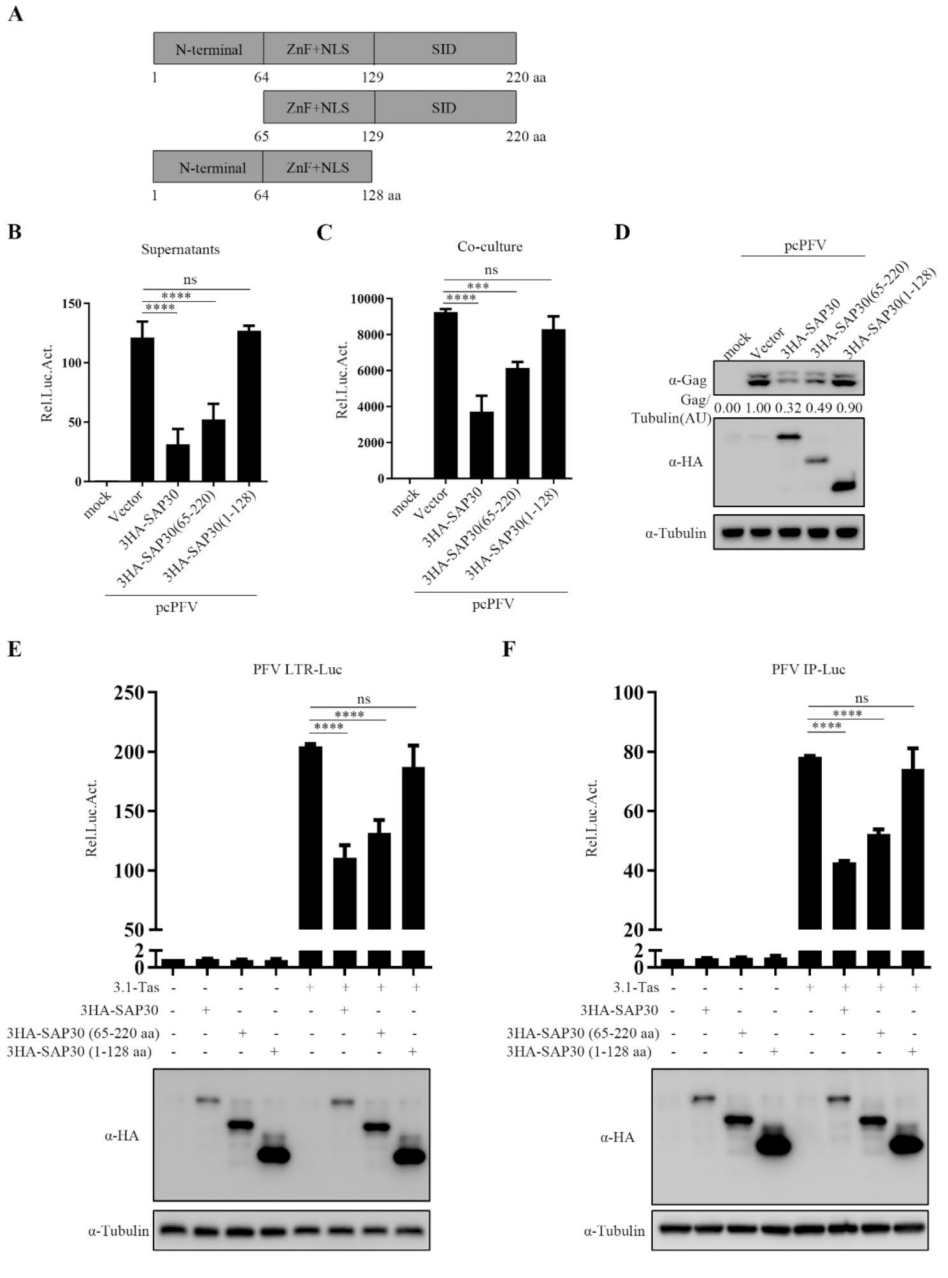
SAP30 Sin3 interaction domain is essential for PFV transcriptional inhibition. (**A**) Truncations of SAP30 protein. (**B**–**D**) HT1080 cells were transfected with pcPFV and empty vector or SAP30 and different truncations. Forty-eight hours post-transfection, 600 µl of supernatants (**B**) or 1/10 transfected HT1080 cells (**C**) were co-cultured with PFVL cells. The luciferase activity was measured 48 h later, and the remaining HT1080 cells were lysed for Western blotting. Protein levels were quantified by densitometry and normalized to Tubulin levels. AU, arbitrary units (**D**). HT1080 cells were transfected with SAP30 or truncated plasmids and LTR-Luc (**E**) or IP-Luc (**F**) with 3.1-Tas. pCMV-β-gal was also transfected to normalize transfection efficiency. Forty-eight hours post-transfection, luciferase activities were measured and corrected by β-gal catalytic activities. The remaining cell lysates were used for Western blotting

To elucidate the mechanism by which SAP30 inhibits PFV Tas function, we identified the key structural domains of SAP30 responsible for its inhibitory effect on PFV replication. Based on the known functional domains of SAP30 [29], we generated two truncations: SAP30 65–220 aa and SAP30 1–128 aa (Fig. 5A) and evaluated their impact on PFV replication. As shown in Fig. 5B–D, SAP30 65–220 aa retained its inhibitory effect on PFV replication, whereas SAP30 1–128 aa lost this effect. Transcription assays further confirmed that SAP30 65–220 aa suppressed the Tas-mediated transactivation of LTR and IP promoters, while SAP30 1–128 aa had no effect (Fig. 5E and F). These results indicate that the C-terminal 129–220 aa of SAP30, known as the Sin3 interaction domain, is essential for inhibiting PFV Tas protein function.

### SAP30 Sin3 interaction domain mediates PFV Tas deacetylation

**Fig. 6:**
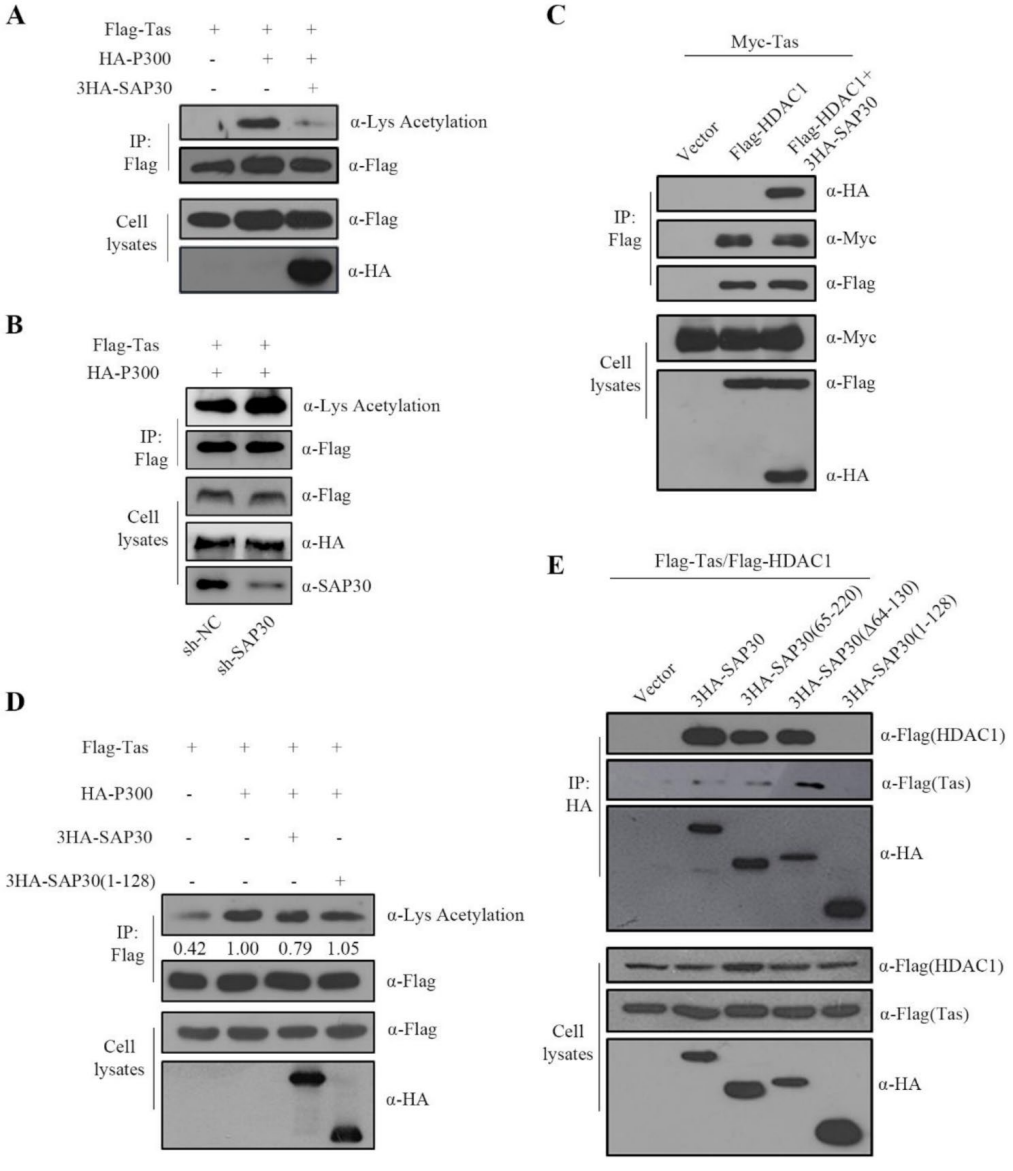
SAP30 Sin3 interaction domain mediates PFV Tas deacetylation. (**A**) HEK293T cells were transfected with Flag-Tas and HA-P300, 3HA-SAP30 or empty vector. Forty-eight hours post-transfection, co-immunoprecipitation was performed with Flag antibodies. Western blot analysis of samples from cell lysates and immunoblotting using Flag, HA and Lys Acetylation antibodies. (**B**) Control and sh-SAP30 cell lines were transfected with Flag-Tas and HA-P300. Forty-eight hours post-transfection, immunoprecipitation was performed with Flag antibodies. Western blot analysis of samples from immunoprecipitates using Flag and Lys Acetylation antibodies. (**C**) HEK293T cells were transfected with Myc-Tas and Flag-HDAC1, 3HA-SAP30 or empty vector. Forty-eight hours post-transfection, co-immunoprecipitation was performed with Flag antibodies. Western blot analysis of samples from cell lysates and immunoprecipitates using Myc, Flag and HA antibodies. (**D**) HEK293T cells were transfected with Flag-Tas, HA-P300 and SAP30 truncations. Forty-eight hours post-transfection, immunoprecipitation was performed with Flag antibodies. Western blot analysis of samples from cell lysates and immunoprecipitates using Flag, HA and Lys Acetylation antibodies. (**E**) HEK293T cells were transfected with Flag-Tas, Flag-HDAC1 and SAP30 truncations. Forty-eight hours post-transfection, co-immunoprecipitation was performed with HA antibodies. Western blot analysis of samples from cell lysates and immunoprecipitates using Flag and HA antibodies

As a core component of the Sin3/HDAC complex, SAP30 binds Sin3 via its Sin3 interaction domain, stabilizing the complex and facilitating HDAC-mediated protein deacetylation, thereby leading to transcriptional repression. Since Tas acetylation is essential for its transactivation of PFV promoters, we examined the effect of SAP30 on Tas acetylation. As shown in Fig. 6A, P300 transfection increased Tas acetylation, whereas SAP30 overexpression significantly reduced it, indicating that SAP30 promotes Tas deacetylation. Conversely, knockdown of endogenous SAP30 increased Tas acetylation levels (Fig. 6B). Co-immunoprecipitation assays further demonstrated interactions among SAP30, HDAC1 and Tas (Fig. 6C), suggesting that SAP30 interacts with Tas to facilitate its deacetylation.

Above experiments demonstrated that the SAP30 Sin3 interaction domain is critical for inhibiting the transactivation function of Tas, and SAP30 can promote the deacetylation of the Tas protein. To determine whether the Sin3 interaction domain plays a key role in Tas deacetylation, we performed immunoprecipitation assays. Deletion of the Sin3 interaction domain abolished SAP30 mediated Tas deacetylation (Fig. 6D). Furthermore, co-immunoprecipitation revealed that deletion of this domain disrupted SAP30 interactions with both Tas and HDAC1 (Fig. 6E). These results suggest that SAP30 interacts with Tas and HDAC1 via its C-terminal 129–220 aa region (Sin3 interaction domain) to mediate Tas deacetylation. This deacetylation ultimately affects Tas transcriptional activity, thereby inhibiting PFV replication.

## Discussion

The persistent infection of FVs within the host is attributed to the interplay between the virus and multiple host genes. In addition to interferon-inducible genes, other host genes may also contribute to PFV replication inhibition in response to PFV stimulation [30–35]. In this study, the novel host gene *SAP30* was identified as an inhibitor of PFV replication. PFV infection upregulated *SAP30* promoter activity through the Tas protein, leading to increased *SAP30* mRNA and protein levels. SAP30 suppresses PFV replication by interacting with the PFV Tas protein and HDAC, thereby promoting Tas deacetylation. The 129–220 aa region of SAP30, known as the Sin3 interaction domain, is critical for this function.

We have been investigating the interaction between PFV and host proteins. In our previous studies, transcriptomic sequencing identified the host proteins SGK1 and PREB, whose mRNA levels were upregulated following PFV infection. We confirmed that PFV increases both the mRNA and protein levels of these proteins, elucidating the molecular mechanisms underlying their effects on PFV replication [26, 36]. Similarly, this study demonstrated that *SAP30* mRNA and protein levels are upregulated after PFV infection and that *SAP30* promoter activity is enhanced in a dose-dependent manner. The PFV Tas protein was identified as the key factor responsible for this enhancement of *SAP30* promoter activity.

The Tas protein is essential for PFV replication and acts as a regulatory switch between latent and lytic infections [37]. Several host proteins have been identified as inhibitors of PFV replication through interactions with Tas. For example, Pirh2 and Trim28 reduce Tas protein expression through interactions, thus inhibiting PFV replication [38, 39], while PML disrupts Tas binding to the viral promoters, suppressing PFV transcription [32]. Our previous studies demonstrated that Nmi, SGK1, and PREB modulate Tas function via interactions, impacting PFV replication [26, 33, 36]. Specifically, Nmi alters Tas intracellular localization, SGK1 affects its transcriptional activation domain, and PREB modulates its DNA binding and transcriptional activation functions. In this study, SAP30 was also found to interact with Tas to influence its function, unlike previous findings, SAP30 inhibited PFV replication by regulating the acetylation levels of the Tas protein.

The acetylation of Tas enhances its transactivation activity on the PFV LTR and IP promoters. Bannert et al. and Bodem et al. demonstrated that the PFV Tas protein interacts with the histone acetyltransferases P300 and PCAF, promoting Tas acetylation and increasing its binding affinity to the promoters, thereby facilitating transcription [12, 13]. Bodem et al. also found that the FFV Tas protein can be acetylated by PCAF [13]. Furthermore, our laboratory previously demonstrated that P300 acetylates the BFV BTas protein, significantly enhancing its transactivation activity [14]. Consistent with these findings, this study further confirms that P300 promotes the acetylation of the PFV Tas protein.

SAP30 is a core component of the human Sin3/HDAC complex and regulates cellular gene transcription by stabilizing the complex. Additionally, SAP30 can directly recruit HDAC to regulate gene expression independently of Sin3. Previous studies have shown that SAP30 influences viral infections, it interacts with PBF to recruit the Sin3/HDAC complex, thereby inhibiting HPV transcription [18], and cooperatively suppresses the transcription of HSV-1-related genes by interacting with HTRP [19]. SAP30 can also be “hijacked” by viruses to enhance infection. For example, the NSs protein of RVFV interacts with SAP30, silencing interferon gene transcription and promoting viral infection [40]. However, no studies have reported an interaction between SAP30 and retroviruses. This study demonstrates that SAP30 interacts with the PFV Tas protein and HDAC via its Sin3 interaction domain, promoting Tas deacetylation, thereby affecting its transcriptional activation function and ultimately inhibiting PFV replication.

In summary, this study identifies SAP30 as a key host factor regulating PFV replication. PFV infection upregulates SAP30 via the Tas protein, which subsequently inhibits PFV replication by promoting Tas deacetylation. This deacetylation impairs Tas mediated transcriptional activation of the PFV LTR and IP promoters, with the Sin3 domain of SAP30 being essential for this inhibitory effect.

## Conclusions

In this study, we found that PFV infection upregulates SAP30 expression. Further investigation revealed that SAP30 inhibits PFV replication and elucidated its specific mechanism. This study not only extends the antiviral spectrum of SAP30 but also contributes to a better understanding of the race game between PFV and the host.

## Data Availability

The sequencing data were deposited at the Gene Expression Omnibus (GEO) repository (accession number is GSE200199).

## Abbreviations

FVs: Foamy viruses
PFV: Prototype foamy virus
FFV: Feline foamy virus
BFV: Bovine foamy virus
LTR: Long terminal repeat
IP: Internal promoter
BTas: Bovine Tas
SAP30: Sin3A associated protein 30
HDAC: Histone deacetylase
PBF: Papillomavirus binding factor
HPV: Human papillomavirus
HSV-1: Herpes simplex virus type 1
HTRP: Human transcription regulator protein
PML: Promyelocytic leukemia
Pirh2: P53-induced RING-H2 protein
Nmi: N-Myc interactor
SGK1: Serum/glucocorticoid regulated kinase 1
PREB: Prolactin regulatory element binding
PFVL: PFV indicator cell line
DEGs: Differentially expressed genes
PPI: Protein-protein interaction
GO: Gene Ontology
